# Supplementary material for: The phosphatidylinositol-transfer protein Nir3 promotes PI(4,5)P2 replenishment in response to TCR signaling during T cell development and survival
Source: Nat Immunol. 2022 Dec 29;24(1):136–47. doi: 10.1038/s41590-022-01372-2 (PMC9810531; doi:10.1038/s41590-022-01372-2)

nature immunology

Article https://doi.org/10.1038/s41590-022-01372-2

The phosphatidylinositol-transfer protein Nir3 promotes $PI(4,5)P_2$ replenishment in response to TCR signaling during T cell development and survival

In the format provided by the
authors and unedited

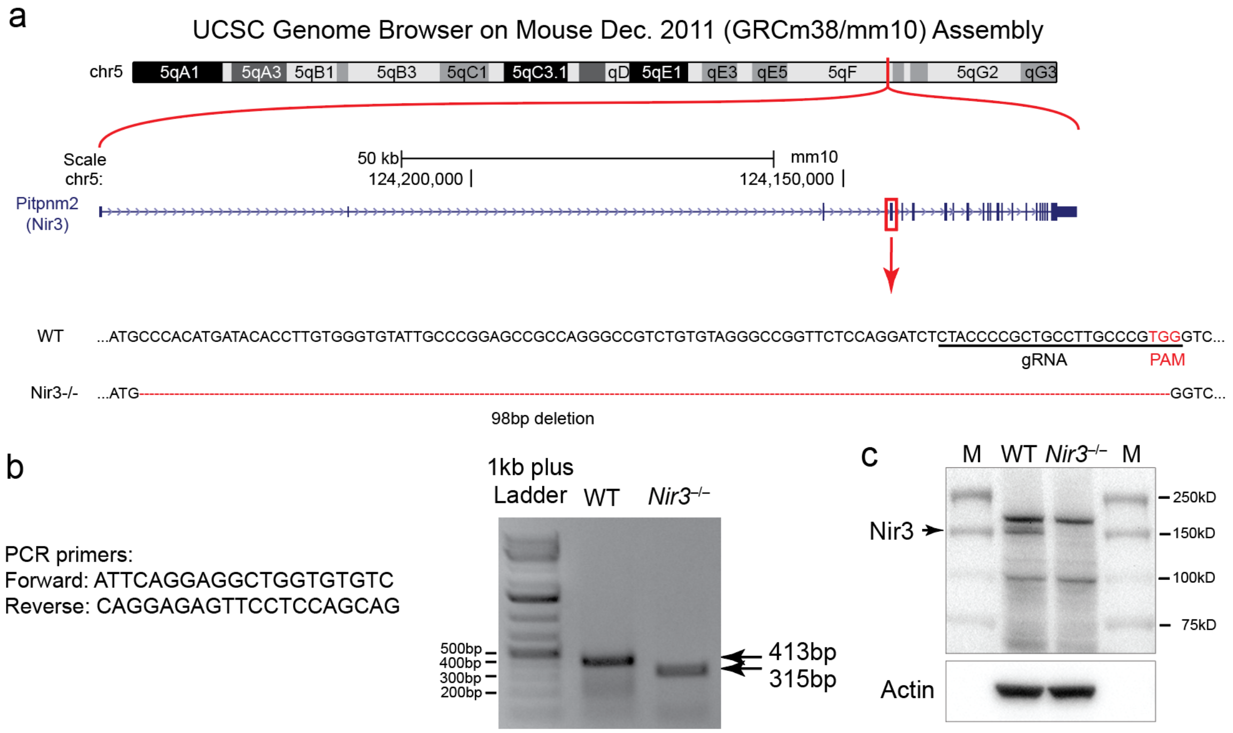

Supplementary Figure 1: Generation of *Nir3*–/– mice. (a) *Nir3* is located on the 5th chromosome in mice. The translation start codon is located on the 3rd exon. A guide RNA (gRNA) targeting site on the 4th exon is underlined and the PAM sequence is marked in red. The gRNA was injected into the C57BL/6 zygotes with the purified Cas9 protein. The resulting pups carried a 98bp deletion on the 4th exon which led to a reading frame shift and introduced a stop codon. (b) *Nir3*–/– mice were confirmed with a primer pair amplifying a region flanking the targeted region. (c) Immunoblot of Nir3 levels from the thymocytes of WT and *Nir3*–/– mice. Actin was used as a loading control. Data are representative of at least 3 separate biological replicates.

Original gels and blots:

b

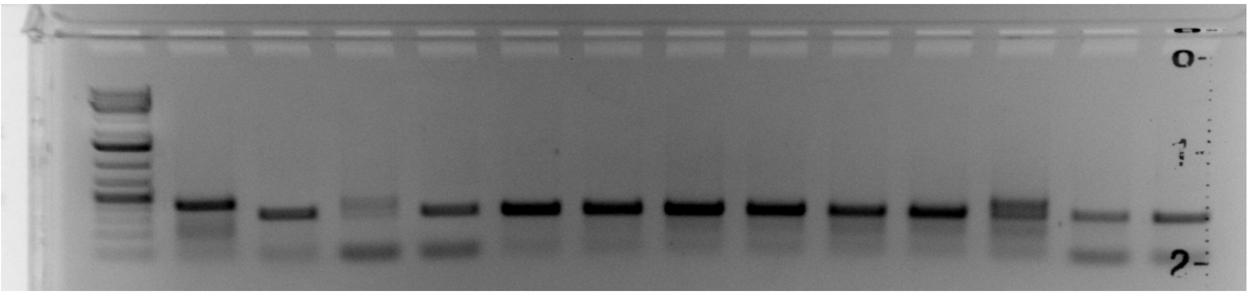

c

Long (left) and short (left) exposure (Upper half: Nir3, Lower half: actin)

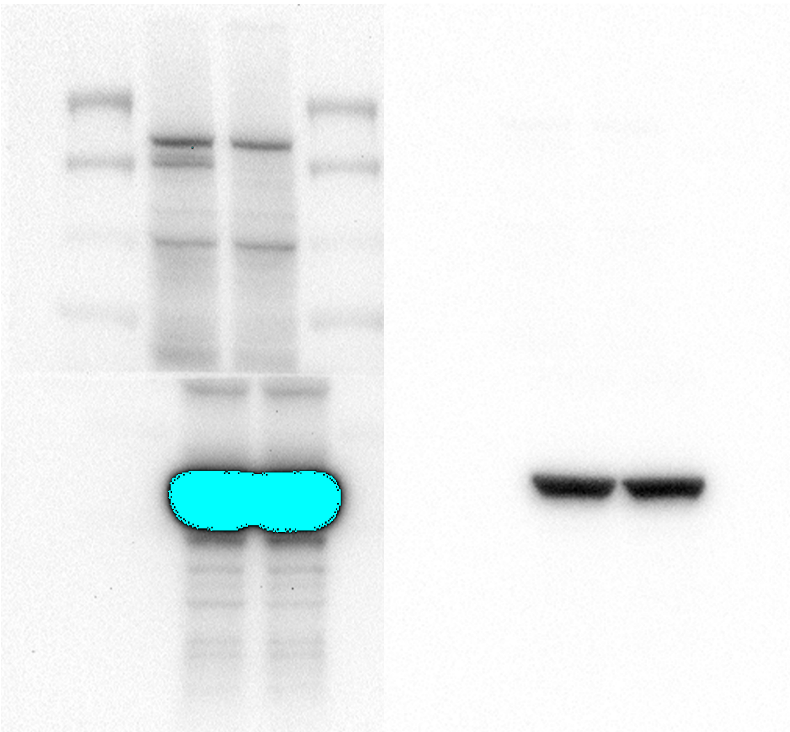

Supplement: Supplementary file 1 — Supplementary Fig. 1. [file 41590_2022_1372_MOESM1_ESM.pdf]
