# Supplementary material for: The phosphatidylinositol-transfer protein Nir3 promotes PI(4,5)P2 replenishment in response to TCR signaling during T cell development and survival
Source: Nat Immunol. 2022 Dec 29;24(1):136–47. doi: 10.1038/s41590-022-01372-2 (PMC9810531; doi:10.1038/s41590-022-01372-2)
Supplement: Supplementary file 2 — Reporting Summary [file 41590_2022_1372_MOESM2_ESM.pdf]

nature portfolio

Corresponding author(s): Arthur Weiss

Last updated by author(s): Oct 17, 2022

Reporting Summary

Nature Portfolio wishes to improve the reproducibility of the work that we publish. This form provides structure for consistency and transparency in reporting. For further information on Nature Portfolio policies, see our Editorial Policies and the Editorial Policy Checklist.

Statistics

For all statistical analyses, confirm that the following items are present in the figure legend, table legend, main text, or Methods section.

n/a	Confirmed	
☐	☒	The exact sample size (*n*) for each experimental group/condition, given as a discrete number and unit of measurement
☐	☒	A statement on whether measurements were taken from distinct samples or whether the same sample was measured repeatedly
☐	☒	The statistical test(s) used AND whether they are one- or two-sided<br>*Only common tests should be described solely by name; describe more complex techniques in the Methods section.*
☒	☐	A description of all covariates tested
☐	☒	A description of any assumptions or corrections, such as tests of normality and adjustment for multiple comparisons
☐	☒	A full description of the statistical parameters including central tendency (e.g. means) or other basic estimates (e.g. regression coefficient) AND variation (e.g. standard deviation) or associated estimates of uncertainty (e.g. confidence intervals)
☐	☒	For null hypothesis testing, the test statistic (e.g. *F*, *t*, *r*) with confidence intervals, effect sizes, degrees of freedom and *P* value noted<br>*Give P values as exact values whenever suitable.*
☒	☐	For Bayesian analysis, information on the choice of priors and Markov chain Monte Carlo settings
☒	☐	For hierarchical and complex designs, identification of the appropriate level for tests and full reporting of outcomes
☒	☐	Estimates of effect sizes (e.g. Cohen's *d*, Pearson's *r*), indicating how they were calculated

Our web collection on statistics for biologists contains articles on many of the points above.

Software and code

Policy information about availability of computer code

Data collection	The flow cytometry data were collected via BD FACSDiva v8.0.1 software. The TIRF images were collected via Nikon NIS-Elements AR imaging software v5.41.02 with A1 and C2 confocal applications
Data analysis	The flow cytometry data were analyzed by BD FLOWJO software version 10.4. In calcium flux experiments, FACS data were exported from FlowJo in CSV format and analyzed with an R script (https://github.com/richard02050411/Calcium-Flux-FACs/blob/master/R-code) by R version 4.0. Graphs and statistics were generated and analyzed using Graphpad Prism software v8.4. The TIRF images were analysis by ImageJ2 v2.3.0/1.53p.<br>In phylogenetic analysis of Nir2 and Nir3, MAFFT was employed to construct a multiple sequence alignment (MSA) for the list of sequences. TrimAl was used to trim for regions with more than 20% gaps and the trimmed MSA was used as input for phylogenetic reconstruction by maximum likelihood analyses through FastTree MP. The initial alignment covered over 750 sequences across 600 species and Phylogenetic Diversity Analyzer was used to generate a sub-sampled sequence list that simultaneously preserved tree features but with an emphasis on the vertebrate lineage with select but well-spaced outgroups including chordates, invertebrates, and sponges (totally 200 sequences). The sub-sampled list was subsequently aligned, trimmed, and a tree was generated as described above, and annotated manually using the ITOL website.

For manuscripts utilizing custom algorithms or software that are central to the research but not yet described in published literature, software must be made available to editors and reviewers. We strongly encourage code deposition in a community repository (e.g. GitHub). See the Nature Portfolio guidelines for submitting code & software for further information.

Data

Policy information about availability of data

All manuscripts must include a data availability statement. This statement should provide the following information, where applicable:

- Accession codes, unique identifiers, or web links for publicly available datasets
- A description of any restrictions on data availability
- For clinical datasets or third party data, please ensure that the statement adheres to our policy

RNA expression profiles of Nir2 and Nir3 in human tissues and mouse T cell populations are available on The Human Protein Atlas (https://www.proteinatlas.org/) and Immunological Genome Project (https://www.immgen.org/), respectively.

Field-specific reporting

Please select the one below that is the best fit for your research. If you are not sure, read the appropriate sections before making your selection.

☒ Life sciences ☐ Behavioural & social sciences ☐ Ecological, evolutionary & environmental sciences

For a reference copy of the document with all sections, see nature.com/documents/nr-reporting-summary-flat.pdf

Life sciences study design

All studies must disclose on these points even when the disclosure is negative.

Sample size	For primary mouse cells experiments, cells from the whole organ were used or cell numbers were as indicated in each method section. Cell numbers were decided upon empiricals, and were routinely used in the lab that were known to be sufficient to allow reliable detection of flow cytometry. The animal numbers used in each experiment were indicated in the bar graphs or figure legends. No statistical methods were used to pre-determine sample sizes but our sample sizes are similar to those reported in previous publications (see methods for references).
Data exclusions	We did not exclude any sample.
Replication	The findings were reliably reproducible. Each set of experiments was repeated at least for two times as indicated in the figure legends. Different experiments were designed to test the hypothesis from orthogonal perspectives. For T cell developmental experiments, we utilized two different approaches (CRISPR knockout animals and bone marrow chimera animals) and obtained similar results.
Randomization	We did not use randomization in our experiments. In animal experiments, age matched animals were allocated based on their genotypes. In cell stimulation experiments, cells with the same genotype were pulled together and equally allocated into groups before receiving different stimulations.
Blinding	Data collection and analysis were not performed blind to the conditions of the experiment. For T cell development experiments, group allocation was not applicable because mice were grouped based on and compared across different genotypes.

Reporting for specific materials, systems and methods

We require information from authors about some types of materials, experimental systems and methods used in many studies. Here, indicate whether each material, system or method listed is relevant to your study. If you are not sure if a list item applies to your research, read the appropriate section before selecting a response.

Materials & experimental systems

n/a	Involved in the study	
☐	☒	Antibodies
☐	☒	Eukaryotic cell lines
☒	☐	Palaeontology and archaeology
☐	☒	Animals and other organisms
☒	☐	Human research participants
☒	☐	Clinical data
☒	☐	Dual use research of concern

Methods

n/a	Involved in the study	
☒	☐	ChIP-seq
☐	☒	Flow cytometry
☒	☐	MRI-based neuroimaging

Antibodies

Antibodies used	Antibody name (clone, source, catalogue number, working dilution)<br>Nir2 Polyclonal antibody (Rabbit polyclonal, Proteintech, 26983-1-AP, 1:1000)<br>PITPNM2 / NIR3 Antibody (aa162-435) (Rabbit polyclonal, Lifespan Bio, LS-C178853, 1:1000)<br>Biotinylated Anti-PtdIns(4,5)P2 IgM (2C11, Echelon-inc, z-b045, 1:100)

AffiniPure Goat Anti-Armenian Hamster IgG (H+L) (Jackson ImmunoResearch Lab, 127-005-099, 1:100)
BD Pharmingen™ PE Rat anti-Mouse Foxp3 (MF23, BD Biosciences, 560408, 1:100)
Armenian Hamster anti-mouse CD3ε Antibody (2c11, Weiss lab, 1:100)
BD Pharmingen™ APC Rat Anti-Mouse CD5 (53-7.3, BD Biosciences, 550035, 1:100)
BD Horizon™ BUV395 Rat Anti-Mouse CD4 (RM4-4, BD Biosciences, 563790, 1:100)
BD Pharmingen™ FITC Rat Anti-Mouse CD8a (53 6.7, BD Biosciences, 553030, 1:100)
BD Horizon™ BUV737 Rat Anti-Mouse CD8a (53 6.7, BD Biosciences, 612759, 1:100)
APC-CD1d-PBS-57 tetramer (NIH tetramer core , 1:100)
BD Horizon™ PE-CF594 Mouse Anti-PLZF (R17-809, BD Biosciences, 565738, 1:100)
BD Pharmingen™ PE Mouse anti-Mouse RORγt (Q31-378, BD Biosciences, 562607, 1:100)
BV605-Armenian monoclonal antimouse CD69 (H1.2F3, BD Biosciences, 563290, 1:100)
PE-Cy7-rat monoclonal antimouse CD24 (M1/69, BD Biosciences, 560536, 1:100)
rabbit monoclonal antimouse/human phopspho-p44/42 MAPK (Erk1/2) (Thr202/Tyr204) (197G2, Cell Signaling Technology, 4376, 1:100)
BD Horizon™ BV421 Mouse Anti-Mouse CD45.1 (A20, BD Biosciences, 563983, 1:100)
BD Pharmingen™ PE-Cy™7 Mouse Anti-Mouse CD45.2 (104, BD Biosciences, 560696, 1:100)
BV711-anti-mouse TCR β chain Antibody (H57-597, BD Biosciences, 563135, 1:100)
BV421-anti-mouse TCR β chain Antibody (H57-597, BD Biosciences, 562839, 1:100)
BD Horizon™ BUV737 Rat Anti-Mouse CD5 (53-7.3, BD Biosciences, 612809, 1:100)
PE-anti-mouse/rat/human CD27 Antibody (LG.3A10, BD Biosciences, 558754, 1:100)
BV605-anti-mouse CD25 Antibody (PC61, BD Biosciences, 563061, 1:100)
PE-Cy™7 Rat Anti-Mouse CD44 (IM7, BD Biosciences, 560569, 1:100)
BV605-anti-mouse CD62L Antibody (MEL-14, BD Biosciences, 563252, 1:100)
BD Horizon™ BV786 Streptavidin (BD Biosciences, 563858, 1:100)
BD Horizon™ PE-CF594 Mouse Anti-Ki-67 (B56, BD Biosciences, 567120, 1:100)
BD Horizon™ PE-CF594 Rat Anti-Mouse CD122 (TM-β1, BD Biosciences, 564763, 1:100)
Nur77 Monoclonal Antibody (12.14), PE, eBioscience™ (12.14, ThermoFisher Scientific, 12-5965-82, 1:100)

Validation

All the antibodies are from commercial sources and have been validated by the vendors. Validation data are available on the manufacturer's website as well as summarized in supplementary table 2.

Eukaryotic cell lines

Policy information about cell lines

Cell line source(s)	HEK293 cells were obtained form UCSF cell culture facility.
Authentication	HEK293 cells were not authenticated
Mycoplasma contamination	The HEK293 cells have been tested for mycoplasma in past years.
Commonly misidentified lines (See ICLAC register)	HEK293 cell line was not listed in ICLAC database.

Animals and other organisms

Policy information about studies involving animals; ARRIVE guidelines recommended for reporting animal research

Laboratory animals	Nir3 deficient mice were generated as described in the method section and backcrossed to C57BL/6 mice for at least six generations. BoyJ (CD45.1) mice (B6.SJL-Ptprca Pepcb/BoyJ) and OT-1 mice (C57BL/6-Tg(TcraTcrb)1100Mjb/J) were originally obtained from The Jackson Laboratory . CD45.1/CD45.2 mice were generated by crossing BoyJ and C57BL/6 mice. All mice used in these studies were housed in a specific pathogen-free facility at UCSF according to the University Animal Care Committee and National Institutes of Health (NIH) guidelines. The temperature of the mouse room is between 68-79°F with 30-70% humidity. A 12 light/12 dark cycle is used.
Wild animals	The study did not involve wild animals.
Field-collected samples	The study did not involve samples collected from the field.
Ethics oversight	Animals were maintained in accordance with Institutional Animal Care and Use Committee protocols, University of California, or approved by San Francisco veterinary committees, and are in accordance with NIH guidelines.

Note that full information on the approval of the study protocol must also be provided in the manuscript.

Flow Cytometry

Plots

Confirm that:

☒ The axis labels state the marker and fluorochrome used (e.g. CD4-FITC).

☒ The axis scales are clearly visible. Include numbers along axes only for bottom left plot of group (a 'group' is an analysis of identical markers).

☒ All plots are contour plots with outliers or pseudocolor plots.

☒ A numerical value for number of cells or percentage (with statistics) is provided.

Methodology

Sample preparation	Thymus, spleens and lymph node single cell suspensions were prepared by gently tweezing samples in cold PBS buffer containing 0.5% BSA and 0.2% EDTA.
Instrument	BD LSR Fortessa was used to collect flow cytometry data.
Software	BD FACSDiva v8.0.1 software was used to collect samples. FlowJo v10.4 was used to analyze flow cytometry data.
Cell population abundance	Sorted DP1 and DP5 thymocytes were about 90% purity.
Gating strategy	Doublets were excluded using forward light-scatter gating followed by gating on lymphocytes based on FSC-SSC. Dead cells were excluded by live/dead stain. Additional gating strategies were provided in the figures (Fig. 2a, 2d, 2e, 2h, 3a, 3c, 3e, 4a, 4b, 4e, 4h and 5f)

☒ Tick this box to confirm that a figure exemplifying the gating strategy is provided in the Supplementary Information.
