# Supplementary figures and images for: The phosphatidylinositol-transfer protein Nir3 promotes PI(4,5)P2 replenishment in response to TCR signaling during T cell development and survival
Source: Nat Immunol. 2022 Dec 29;24(1):136–47. doi: 10.1038/s41590-022-01372-2 (PMC9810531; doi:10.1038/s41590-022-01372-2)

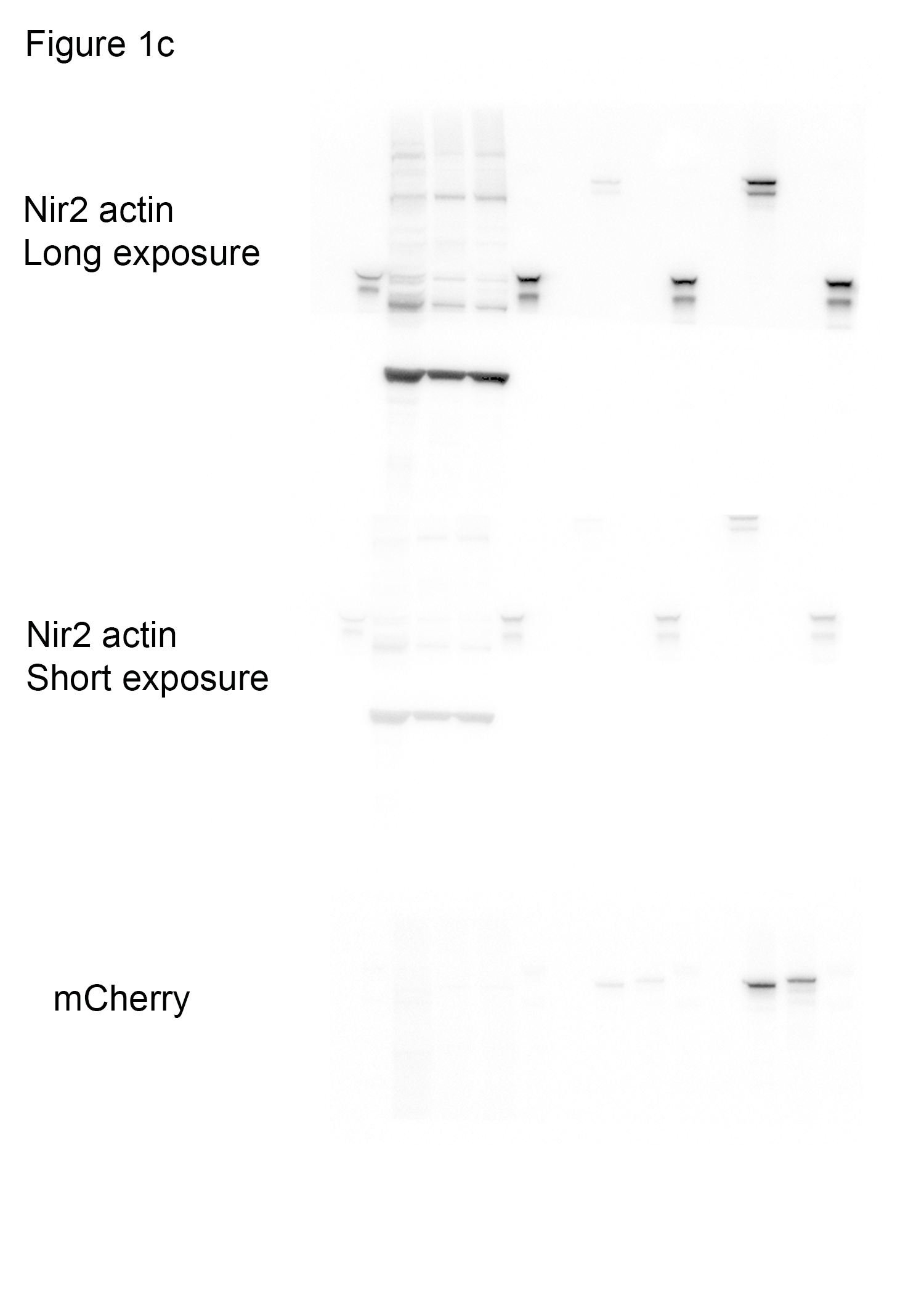

Supplement: Source Data Fig. 1 — Unprocessed western blots. [file 41590_2022_1372_MOESM6_ESM.tif]

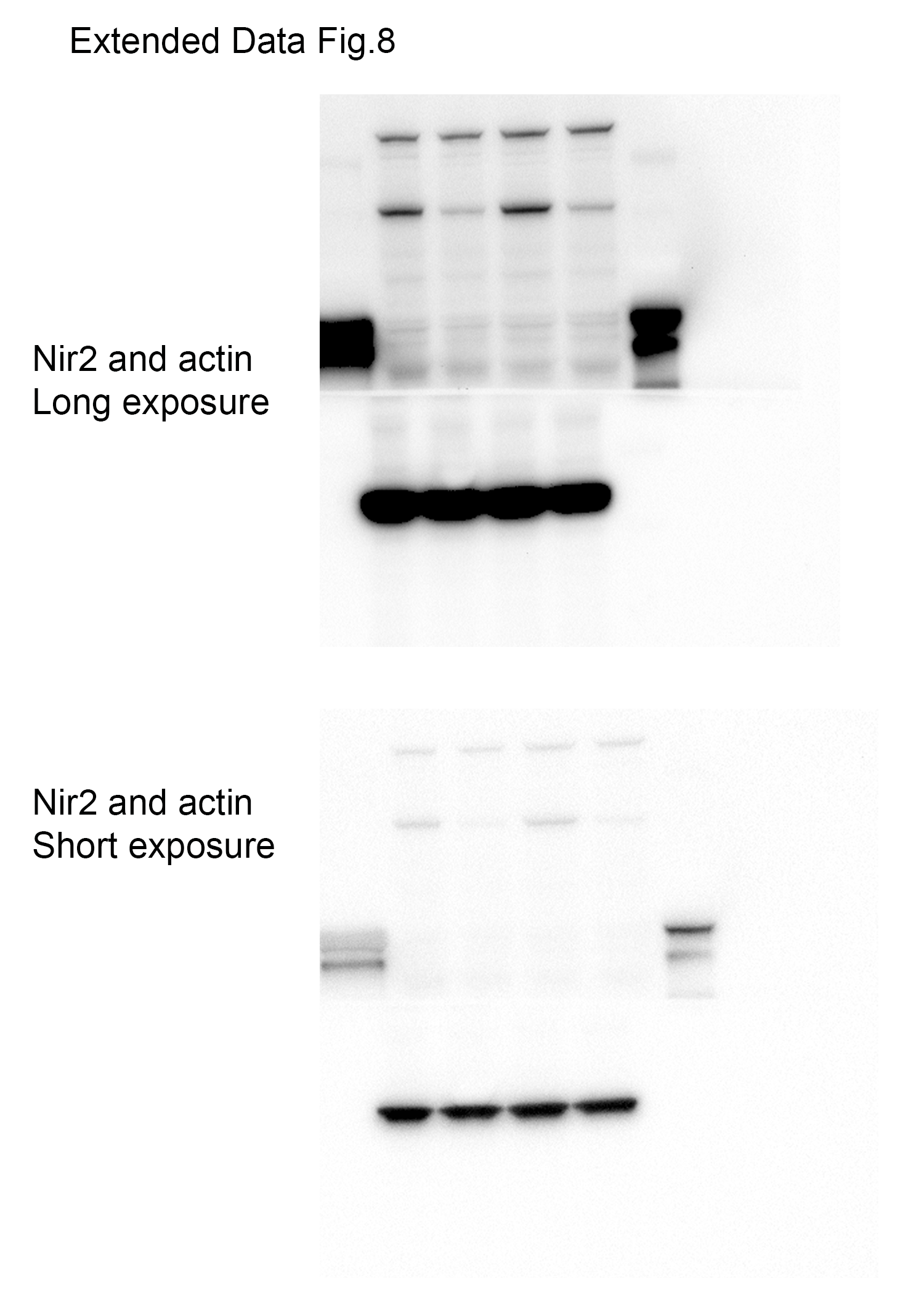

Supplement: Source Data Extended Data Fig. 8 — Unprocessed western blots. [file 41590_2022_1372_MOESM22_ESM.tif]
